# Assessment of urinary kidney injury molecule-1 and interleukin-18 in the early post-burn period to predict acute kidney injury for various degrees of burn injury

**DOI:** 10.1186/s12882-015-0140-3

**Published:** 2015-08-18

**Authors:** Hongqi Ren, Xuan Zhou, Deshu Dai, Xiang Liu, Liangxi Wang, Yifang Zhou, Xiaomei Luo, Qing Cai

**Affiliations:** Department of Nephrology, Jinling Hospital, Nanjing Clinical College of Second Military Medical University, 305 Zhongshan East Road, Nanjing, China; Department of Nephrology, HuaiHai Hospital of Xuzhou Medicine College, 226 Tongshan Road, XuZhou, 221004 China; Department of Burns, HuaiHai Hospital of Xuzhou Medicine College, 226 Tongshan Road, XuZhou, 221004 China; Department of Clinical Laboratory, HuaiHai Hospital of Xuzhou Medicine College, 226 Tongshan Road, XuZhou, 221004 China

**Keywords:** Burns, Interleukin-18, Acute kidney injury, Kidney injury molecule-1

## Abstract

**Background:**

Burn patients with AKI have a higher mortality, rapid diagnosis and early treatment of AKI are necessary. Recent studies have demonstrated that urinary KIM-1 and IL-18 are potential biomarkers of early-stage AKI, however, changes in urinary KIM-1 and IL-18 levels are unclear in patients with burns. The aim of our study was to determine whether combined KIM-1 and IL-18 are more sensitive than traditional markers in detecting kidney injury in patients with burns.

**Methods:**

Ninety-five burn patients hospitalized at the Burns and Plastic Surgery Center of our hospital from April 2013 to September 2013 were enrolled into this prospective study and divided into mild- (*n* = 37), moderate- (*n* = 30) and severe-burn groups (*n* = 28) by burn injury surface area. In the moderate- and severe-burn groups, patients were subcategorized to either the acute kidney injury (AKI) group, in which serum creatinine (Scr) increased to ≥26.5 μmol/L within 48 h, or the non-AKI group. Fifteen healthy subjects were selected as a control group. Blood specimens were collected to determine blood urea nitrogen (BUN), Scr, and other biochemical indicators. Urine samples collected at admission and 48 h after admission were analyzed for KIM-1 and IL-18. Correlations among urinary KIM-1 and IL-18, burn degree, and clinical biochemical indicators were investigated.

**Results:**

AKI occurred in 11.2 % of burn patients (none in the mild-burn group). AKI developed 48 h after admission in 10.0 % of the moderate- and 28.6 % of the severe-burn groups. Urinary KIM-1 concentration in the moderate- and severe-burn groups was significantly higher than that in the control group; urinary IL-18 concentrations did not differ significantly among the burn and control groups. The AKI group had significantly higher concentrations of urinary KIM-1 and IL-18 than the non-AKI group, both at admission (*p* = 0.001 and *p* < 0.001, respectively) and 48 h later (*p* = 0.001 and *p* < 0.001, respectively). Both urinary KIM-1 and IL-18 increased before Scr. Receiver operating-curve (ROC) analysis demonstrated that KIM-1 combined with IL-18 predicted AKI with 72.7 % sensitivity and 92.8 % specificity. The area under the ROC curve was 0.904.

**Conclusions:**

Our results suggest that urinary KIM-1 and IL-18 may be used as early, sensitive indicators of AKI in patients with burns of varying degrees and provide clinical clues that can be used in early prevention of AKI.

## Background

Acute kidney injury (AKI), a common and serious complication of large-area burns, is a critical condition clinically characterized by oliguria and elevated serum creatinine (Scr) [1]. The incidence of AKI is 45.5 %; severe AKI occurs in 0.5 to 30 % of burn patients and is associated with the size and severity of the burn [1, 2]. Patients with AKI tend to have higher mortality than those without AKI; in particular, the mortality associated with burns and severe AKI is greater than 80 % [2–4]. Therefore, early diagnosis of AKI is important to undertaking effective treatment [5].

According to the Kidney Disease Improving Global Outcomes (KDIGO) 2012 AKI guidelines, AKI is defined as an increase in Scr to ≥26.5 μmol/L within 48 h, or to ≥1.5 times the baseline value within 7 days. However, the guidelines use urine output and Scr values as staging criteria. Moreover, because Scr is affected by many factors and has low sensitivity and specificity for an association with AKI, it is particularly important to determine other biomarkers of early kidney injury that are sensitive, specific, and reliable. Numerous studies conducted in recent years have indicated that factors such as kidney injury molecule-1 (KIM-1), interleukin-18 (IL-18), N-acetyl-β-glucosidase (NAG) and neutrophil gelatinase-associated lipocalin (NGAL) increase in the early stages of AKI and can therefore be used as markers for early AKI diagnosis [6–9].

Because burn patients with AKI have a higher mortality, rapid diagnosis and early treatment of AKI are necessary. Few studies have demonstrated the ability of NGAL to reflect the severity of renal injury or to be used as an indicator of inflammation in burn patients [10] even though plasma and urinary NGAL levels within 48 h after admission have been independently associated with AKI development and mortality [11].

Recent studies have demonstrated that urinary KIM-1 and IL-18 are potential biomarkers of early-stage AKI caused by contrast-induced nephrotoxicity, cardiac surgery, and preeclampsia [12–14], and can reveal AKI much earlier than canScr. However, changes in urinary KIM-1 and IL-18 levels are unclear in patients with burns. The aim of our study was therefore to determine whether combined KIM-1 and IL-18 are more sensitive than traditional markers in detecting kidney injury in patients with burns.

## Methods

### Patients

One hundred thirty-one burn patients hospitalized at the Burns and Plastic Surgery Center of our hospital from April 2013 to September 2013 were selected. Exclusion criteria were: 1) age <15 years or >65 years; 2) receiving nephrotoxic drugs prior to admission; 3) previous history of heart failure, coronary heart disease, chronic liver disease, or end-stage renal disease; 4) the time of burn more than 12 h before admission; and 5) chemical burn. Ultimately, 95 burn patients were enrolled in this study. Patients were divided into mild- (*n* = 37), moderate- (*n* = 30), and severe-burn (*n* = 28) groups based on total body surface area (TBSA), burn depth, and complications. Those with second-degree TBSA <10 % were assigned to the mild-burn group, and those with second-degree TBSA of approximately 11–30 % or third-degree TBSA <10 % were assigned to the moderate-burn group. Those with second-degree TBSA exceeding 31 % with third-degree TBSA >10 %, or whose second- or third-degree TBSA had not yet reached the previously defined percentages but who had shock or other complications, including respiratory burns or severe combined injuries, were assigned to the severe-burn group [2]. Additionally, 15 healthy volunteers from the medical center of our hospital over the same period were selected as the control group.

Clinical data, including age, sex, third-degree-burn area, presence of inhalation injury, presence of rhabdomyolysis, and APACHE II score on admission and 48 h after admission, were collected from all patients. A patient was diagnosed with AKI if Scr had increased to meet the AKI criteria according to KDIGO guidelines by 48 h after admission.

This study was approved by the Ethical Review Board of the HuaiHai Hospital of Xuzhou Medicine College,China. Each participant provided written informed consent for participation in the study; however, any participant who could not personally complete the informed-consent form did so with the assistance of his or her parent or a relative.

### Collection, preservation, and testing of urine specimens

Clean-catch midstream urine specimens were collected from patients in each group at the time of admission and were collected again 48 h after admission. Patients with compromised mental status were given an indwelling catheter to obtain clean urine. After centrifugation for 5 min at 3,000 rpm, the supernatant was transferred into Eppendorf tubes and stored in a freezer at −80 °C. Urine specimens for the control group were also collected and stored at −80 °C.

Urinary KIM-1 and IL-18 levels were measured by enzyme-linked immunosorbent assay (ELISA) (Human TIM-1/KIM-1/HAVCR Quantikine ELISA Kit, R&D Systems, Minneapolis, MNUSA; Human IL-18 Platinum ELISA, eBioscience, Inc, San Diego, CA, USA). Optical density was read at 450 nm (Model 450 Microplate Reader; Bio-Rad Laboratories, Inc., Hercules, CA, USA), and the test-sample concentrations were calculated using a standard curve.

### Blood-specimen testing

Blood samples were also collected at, and 48 h after, the time of admission. After routine blood examination, a model 7000 Automatic Biochemical Analyzer (Hitachi High Technologies America, Inc., Schaumberg, IL, USA) was used to determine Scr as well as serum alanine aminotransferase (ALT), aspartate aminotransferase (AST), albumin (Alb), blood urea nitrogen (BUN), and creatine kinase (CK).

### Statistical analysis

PASW Statistics for Windows, Version 18.0 (SPSS Inc., Chicago, IL, USA) was used for statistical analyses. Results are expressed as mean ± standard deviation, median and interquartile range, or number and percentage. Student’s *t*-test was applied to parametric data and the Mann–Whitney *U-* test to nonparametric data. Inter-group comparisons were performed using analysis of variance. The chi-square test was applied to data expressed as a rate. Pearson correlation analysis was also used. The diagnostic abilities of urinary KIM-1 and IL-18 levels to predict AKI were assessed by calculating the areas under the receiver operating characteristic (ROC) curves (AUC) and the cutoff value was defined by Youden’s index.

In addition, Stata 13.0 statistical software (StataCorp LP, College Station, TX, USA) was used for the Net Reclassification Improvement (NRI) and Integrated Discrimination Improvement (IDI) analyses for KIM-1 and IL-18.

Differences with *p* values <0.05 were considered statistically significant.

## Results

Table 1 presents clinical data, including TBSA calculation, laboratory results, and burn-group assignments performed at admission, for the 95 study patients.

**Table 1:** Patient data for the control group and burn groups

	Control (*n* = 15)	Mild burn (*n* = 37)	Moderate burn (*n* = 30)	Severe burn (*n* = 28)	*p* value
M/F	11/4	29/8	23/7	23/5	0.481
Age (y)	38.5 ± 10.2	31.5 ± 15.5	35.7 ± 18.4	40.6 ± 14.8	0.121
TBSA (%)	/	7.01 ± 1.61	24.9 ± 3.48	44.4 ± 13.8	/
BUN (mmol/L)	4.14 ± 0.87	4.91 ± 1.53	4.79 ± 1.71	4.88 ± 0.88	0.161
Scr (μmol/L)	59.0 ± 8.73	54.6 ± 20.8	60.9 ± 20.9	60.9 ± 18.4	0.476
Urinary KIM-1 (ng/mL)	2.12 ± 0.80	2.65 ± 0.73	4.06 ± 1.38^ab^	5.06 ± 1.51^abc^	0.000
Urinary IL-18 (pg/mL)	6.04 ± 1.41	6.27 ± 2.84	6.93 ± 3.11	6.68 ± 2.66	0.671

Data expressed as mean ± standard deviation unless otherwise indicated. Alb, albumin; BUN, blood urea nitrogen; F, female; IL-18, interleukin-18; KIM-1, kidney injury molecule-1; M, male; Scr, serum creatinine; TBSA, total burn surface area

^a^vs. control group; ^b^vs. mild-burn group; ^c^vs. moderate-burn group

### Laboratory parameters and urinary KIM-1 and IL-18 levels

There were no significant differences in Scr or BUN levels among the mild-, moderate-, and severe-burn and control groups at admission (Table 1).

Urinary KIM-1 levels increased with burn severity, and significant differences were observed among groups. Urinary IL-18 levels did not differ significantly among groups (Table 1).

### Development of AKI in burn patients

**Fig. 1:**
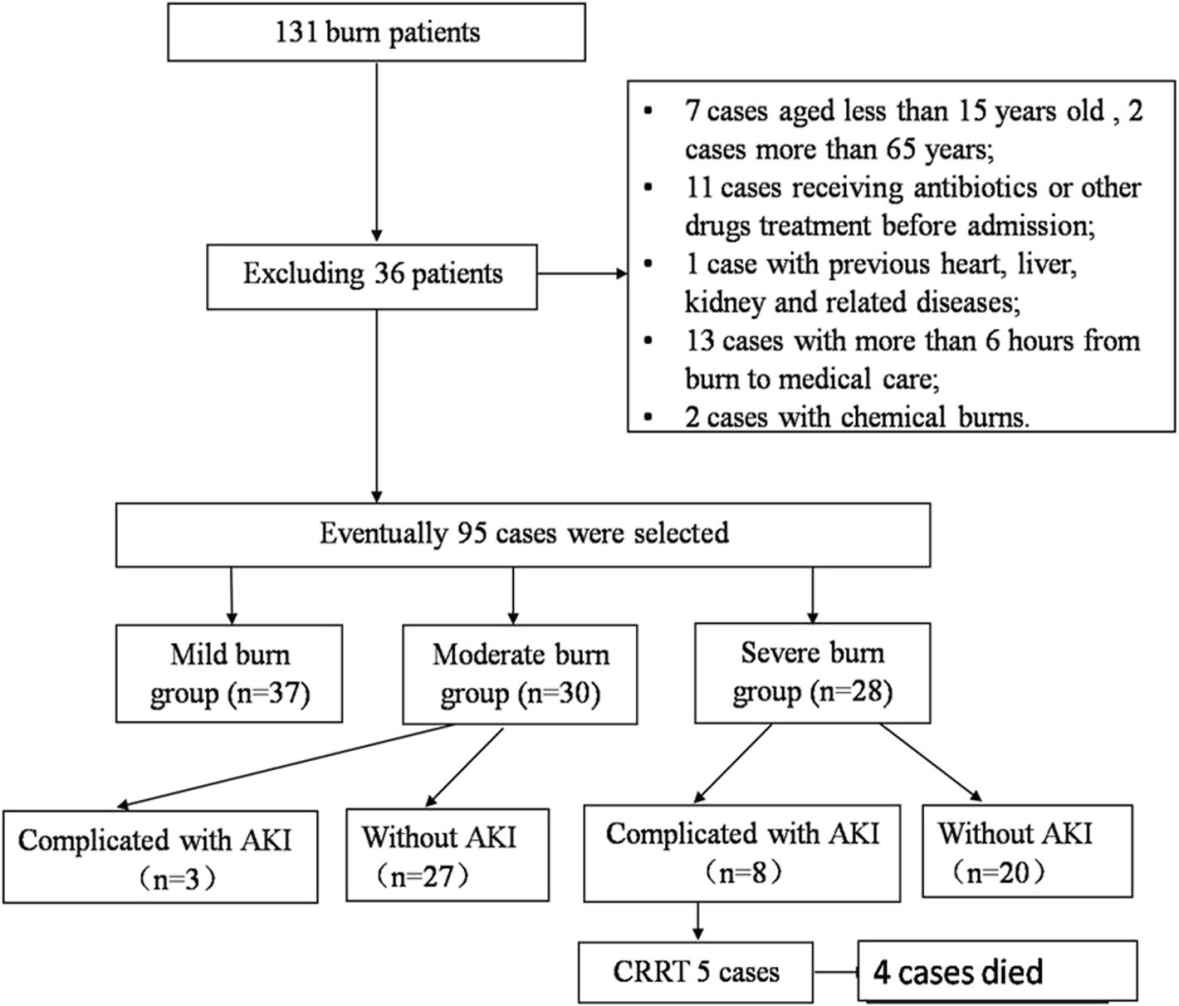
Flowchart of patient selection. AKI, acute kidney injury; CRRT, Continuous renal replacement therapy

The incidence of AKI in burn patients within 48 h after admission was 11 of 95 patients (11.2 %). No patients in the mild-burn group, three of the 30 patients in the moderate-burn group (10 %), and eight of 28 patients in the severe-burn group (28.6 %) developed AKI within 48 h after admission. The incidence of AKI in the severe-burn group was significantly higher than that in the moderate-burn group (Fig. 1). These 11 of 58 patients with moderate and severe burns were sub-categorized to the AKI group (seven males, four females; age, 33.4 ± 11.3 years). The KDIGO Guidelines on AKI were used to classify five of the 11 as stage 2, five as stage 3, and one as stage 1 AKI. The other patients in the moderate- and severe-burn groups were sub-categorized to the non-AKI group.

### Comparisons between AKI and non-AKI groups

We observed significant differences in TBSA, third-degree burn area (TDBA), proportion of patients with inhalation injury, and incidence of rhabdomyolysis between the AKI and non-AKI groups at the time of admission (Table 2).

**Table 2:** Clinical features and laboratory results of non-AKI and AKI groups

	Non-AKI group (*n* = 47)	AKI group (*n* = 11)	*p* value
At the time of admission			
Age (y)	39.1 ± 17.7	33.4 ± 11.3	0.309
gender (M/F)	39/8	7/4	0.154
TBSA (%)	32.0 ± 10.1	44.2 ± 22.4^a^	0.008
Third-degree burn area (%)	5(0,14)	27(9,48)	0.000
Inhalation injury,n (%)	6(12.8)	7(63.6)	0.000
Rhabdomyolysis,n (%)	5(10.6)	6(54.5)	0.001
BUN (mmol/L)	4.52 ± 1.14	7.02 ± 1.53	0.000
Scr (μmol/L)	55.2 ± 14.1	85.3 ± 21.6	0.000
Urinary KIM-1 (ng/mL)	4.23 ± 1.25	5.84 ± 1.89	0.001
Urinary IL-18 (pg/mL)	6.15 ± 2.57	9.64 ± 2.43	0.000
APACHE II Score	8.34 ± 5.84	16.3 ± 7.35^a^	0.000
48 h after admission			
BUN (mmol/L)	5.52 ± 1.45	11.4 ± 2.99	0.000
Scr (μmol/L)	57.4 ± 15.0	178.4 ± 58.8	0.000
U KIM-1 (ng/mL)	4.62 ± 1.25	6.18 ± 1.62	0.001
UIL-18 (pg/mL)	5.80 ± 2.16	10.9 ± 2.86	0.000
Mechanical ventilation,n (%)	0	5(45.5)	/
CRRT,n (%)	0	5(45.5)	/
APACHE II Score	8.36 ± 6.38	29.3 ± 7.77^a^	0.000
Wihtin 4 weeks			
Mortality,n (%)	0	4(36.4)	/

APACHE II, Acute Physiology and Chronic Health Evaluation II; BUN, blood urea nitrogen; CRRT, continuous renal replacement therapy; IL-18, interleukin-18; KIM-1, kidney injury molecule-1; Scr, serum creatinine; TBSA, total burn surface area

^a^AKI group vs. non-AKI group

APACHE II score was significantly higher in the AKI group than in the non-AKI group, both at the time of admission and 48 h after admission (Table 2), and was significantly higher 48 h after admission than at the time of admission in the AKI group. In addition, five patients underwent mechanical-ventilation therapy and five were treated with continuous renal replacement therapy (CRRT) during the 48 h after admission. In the AKI group, four patients did not survive more than 4 weeks after admission. Mortality in the AKI group was 36.4 % (Table 2).

### Changes in urinary KIM-1 and IL-18 levels of the AKI non-AKI groups between admission and 48 h after admission

**Fig. 2:**
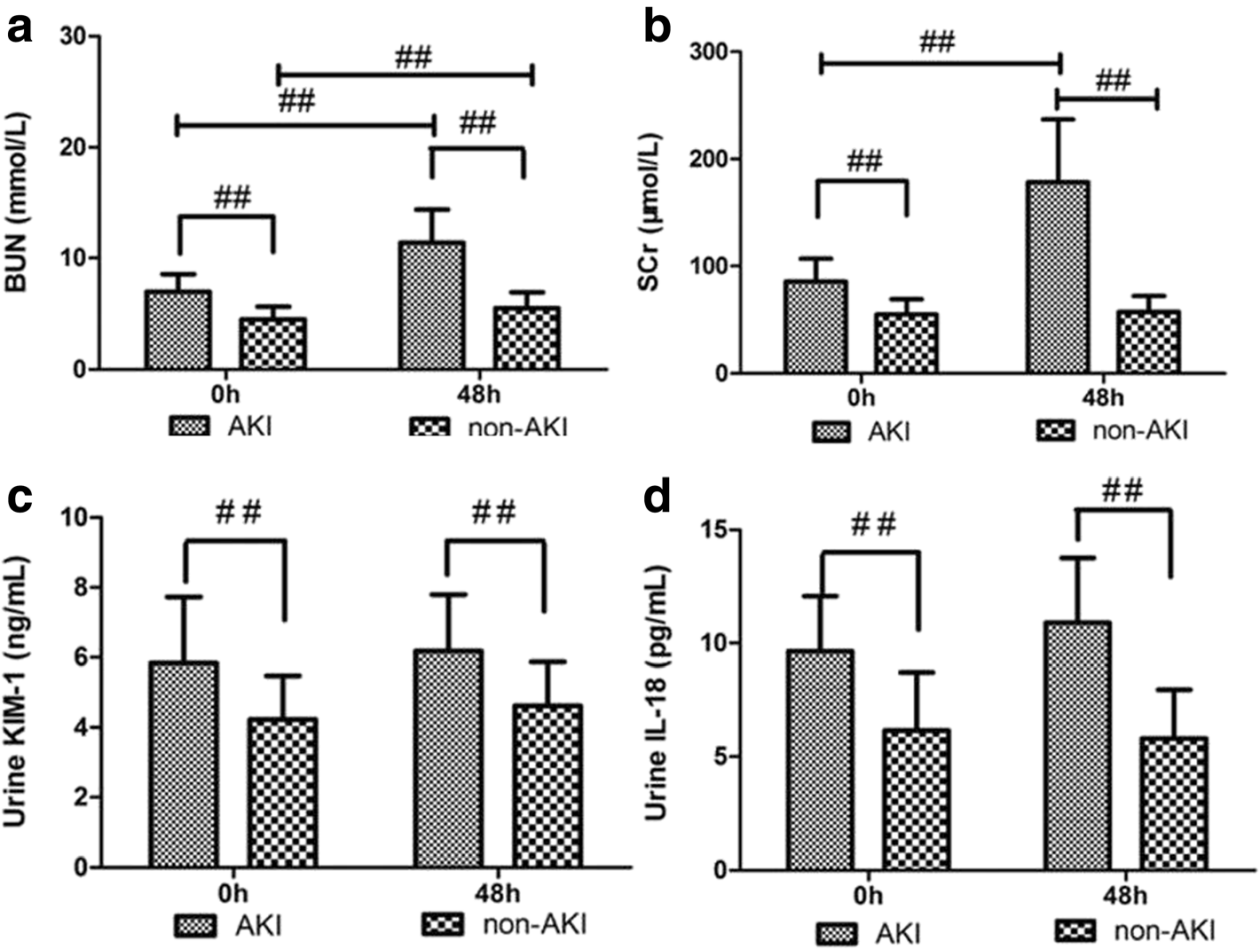
Dynamic changes in urinary KIM-1 and IL-18 levels between AKI and non-AKI groups on admission and 48 h post admission. ^##^ Significant difference (*p* < 0.01) between groups. AKI, acute kidney injury; BUN, blood urea nitrogen; KIM-1, kidney injury molecule-1

Scr and serum BUN levels in the AKI group were significantly higher than those of the non-AKI group, both at the time of admission and 48 h after admission (Table 2), and were significantly higher 48 h after admission than at the time of admission in the AKI group (Fig. 2a, b). However, in the non-AKI group, only BUN was significantly higher at 48 h than at admission (Fig. 2a).

**Fig. 3:**
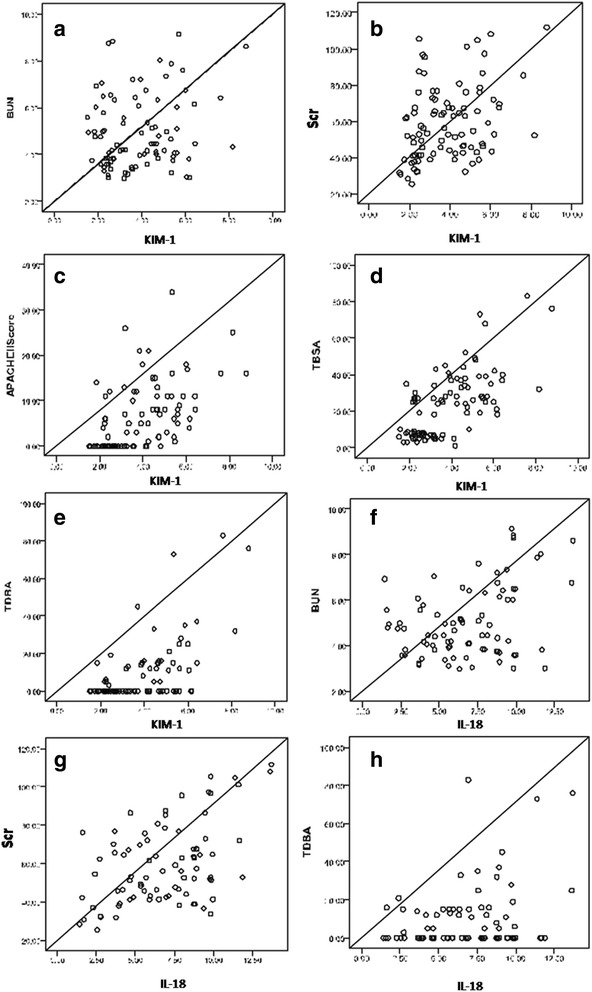
Relationships between urinary KIM-1 levels and **a** serum BUN, **b** Scr, **c** APACHE II Score, **d** TBSA, and **e** TDBA; and between urinary IL-18 levels and **f** serum BUN, **g** Scr, and **h** TDBA. APACHE II, Acute Physiology and Chronic Health Evaluation II; BUN, blood urea nitrogen; KIM-1, kidney injury molecule-1; Scr, serum creatinine; TDBA, third-degree burn area; TBSA, total burn surface area

**Fig. 4:**
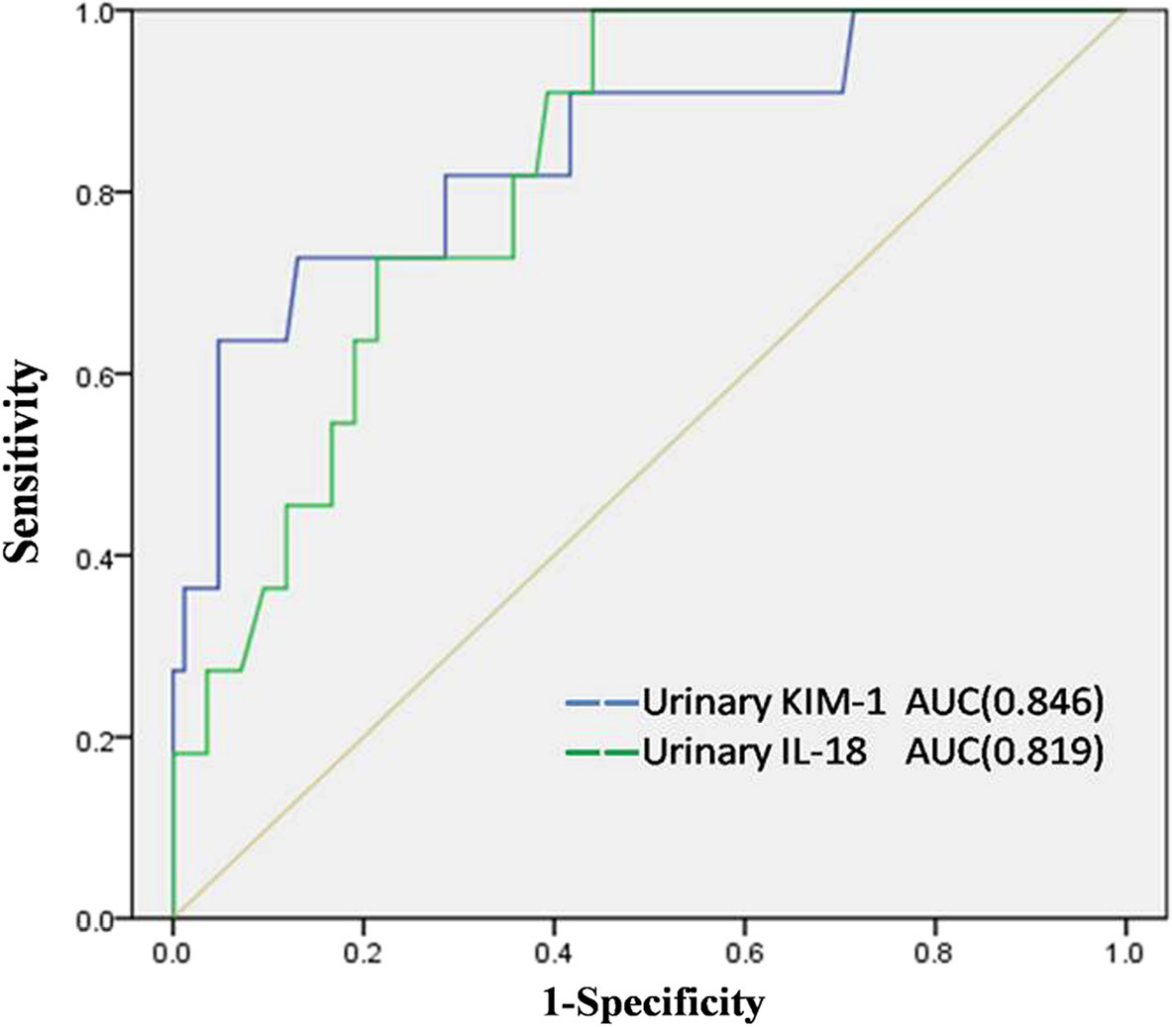
ROC curves showing probability of urinary KIM-1 and urinary IL-18 to predict acute kidney injury on admission. AUC, area under the ROC curve; KIM-1, kidney injury molecule-1; IL-18, interleukin-18; ROC, receiver operating characteristic

Urinary KIM-1 levels were significantly higher in the AKI group than in the non-AKI group both at the time of onset (*p =* 0.001) and 48 h after admission (*p <* 0.001) (Table 2). Levels of urinary KIM-1 and IL-18 did not differ significantly between admission and 48 h after admission in either group (Fig. 2c, d).

### Relationship between urinary KIM-1 and IL-18 and Scr, serum BUN, and other clinical parameters

Correlation analysis between the urinary KIM-1 and IL-18 levels and the clinical indicators of all 95 burn patients was performed (Fig. 3). This demonstrated that urinary KIM-1 at the time of admission was positively correlated with Scr (*r* = 0.358, *p* < 0.001) but not with BUN (*r* = 0.146, *p =* 0.157). Urinary IL-18 at admission was positively correlated with both serum BUN (*r* = 0.344, *p* <0.001) and Scr (r = 0.489, *p* <0.001).

We also analyzed the relationships between urinary KIM-1 and IL-18 and the age, TBSA, TDBA, presence of rhabdomyolysis, and APACHE II scores of burn patients. We observed significant positive correlations between urinary KIM-1 and TBSA (*r* = 0.661, *p* < 0.001), TDBA (*r* = 0.600, *p* <0.001), rhabdomyolysis (*r* = 0.466, *p* <0.001), and APACHE II score (*r* = 0.534, *p* <0.001). However, only urinary IL-18 and TDBA (*r* = 0.307, *p* = 0.002) were seen to be significantly positively correlated. There was no significant correlation between age and either KIM-1 (*r* = 0.079, *p* = 0.449) or IL-18 (*r* = 0.095, *p* = 0.361).

### The power of urinary KIM-1 and IL-18 to predict AKI on admission

**Fig. 5:**
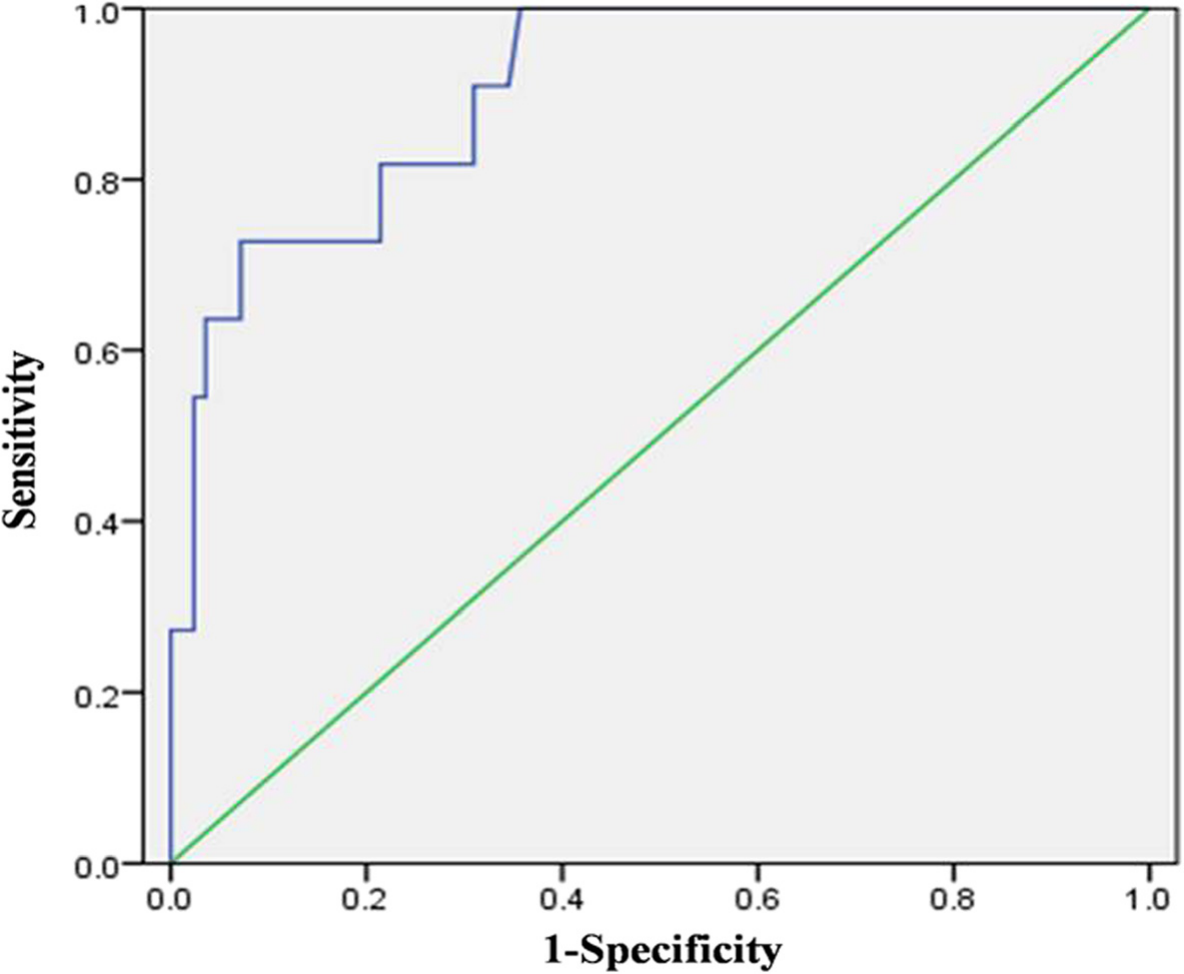
ROC curve showing the probability of the combination of urinary KIM-1 and urinary IL-18 to predict acute kidney injury on admission. The AUC for the combination was 0.904. AUC, area under the ROC curve; IL-18, interleukin-18; KIM-1, kidney injury molecule-1; ROC, receiver operating characteristic

To predict AKI on admission, ROC curves were drawn by plotting sensitivity against 1 − specificity of urinary KIM-1 and IL-18, and the area under curve (AUC) was calculated (Fig. 5). ROC/AUC analysis demonstrated a sensitivity of 72.7 % and a specificity of 86.9 %, (AUC = 0.846; 95 % confidence interval [CI], 0.712–0.980) for a cutoff value of 5.33 ng/mL urinary KIM-1 (Fig. 4). The ROC curve showed the optimal cutoff for urinary IL-18 to be 6.39 pg/mL (AUC = 0.819; 95 % CI 0.714–0.923) with a sensitivity of 100 % and a specificity of 56 % in predicting AKI (Fig. 4). When we combined urinary KIM-1 and IL-18 using the probability function in binary logistic regression, we found that the ROC curve predicting the probability of KIM-1 combined with IL-18 showed a sensitivity of 72.7 % and a specificity of 92.8 % (AUC = 0.904, 95 % CI 0.822–0.987) (Fig. 5).

To estimate the predictive power of each biomarker, we performed NRI and IDI analyses for early AKI and determined the NRI for urinary KIM-1 to be 0.9307 (standard error [SE] = 0.3206, z = 2.9027, *p* = 0.0037), and for urinary IL-18 to be 0.9069 (SE = 0.3206, z = 2.8284, *p* = 0.0047). The IDI for urinary KIM-1 was 0.3068 (SE = 0.0953, z = 3.2180 and *p* = 0.0013); for urinary IL-18 the IDI was 0.1866 (SE = 0.0715, z = 2.6083, *p* = 0.0091).

## Discussion

AKI is characterized as a rapid loss of kidney function that clinically manifests as an abrupt and sustained rise in urea and creatinine. Our prospective study found that the urinary KIM-1 and IL-18 levels may be better diagnostic biomarkers than Scr for early-stage AKI in burn patients.

AKI is a common and serious complication following severe burn injury and is associated with increased morbidity, mortality, and hospitalization costs. An extensive literature review that included approximately 35,000 burn patients found that AKI had a prevalence of 14.5 % and an associated mortality rate of 77.3 % [15]; others have reported that mortality rates among burn patients who develop AKI range from 73 to 100 % [16, 17]. In our study, we found the incidence of AKI, as defined by KDIGO guidelines, among patients in the moderate- and severe-burn groups to be 10.0 and 28.6 %, respectively, and the mortality rate of burn patients with AKI to be 36.4 %. The high likelihood of developing AKI after burn injury greatly increases mortality among burn patients, particularly those with severe burns complicated by AKI. Therefore, early identification of patients at risk for AKI is important.

Although Scr is used routinely in clinical practice, it may be a suboptimal marker of acute renal injury. Increased Scr is related more to loss of filtration function than to acute tubular injury [18]. Because a detectable increase in Scr is delayed, it is not a reliable early biomarker for AKI. Therefore, sensitive, specific, and reliable biomarkers are urgently needed. Increases in the expression of urinary KIM-1 and IL-18 and other biological markers have recently been reported to occur within 2 h of renal injury and gradually approach their peaks [9, 19]. The aim of our study was therefore to choose the markers of acute renal tubular damage to diagnose AKI. Because some studies have shown that use of single biomarkers may not be adequate to determine whether AKI is due to kidney heterogeneity or various dysfunctions, we combined KIM-1 with IL-18 and clinical observations for early diagnosis of AKI in burn patients and rapid therapeutic interventions.

Urinary KIM-1 may be one of the most stable, reliable, sensitive, and specific indicators for early diagnosis of AKI. KIM-1, a type I transmembrane protein, was identified in 1998 by Lim et al in a study of ischemia-reperfusion in rat kidney cells using representational difference analysis [20, 21]. Han et al [22] were the first to detect the significant expression of KIM-1 in humans using renal biopsy specimens of patients with acute tubular necrosis. Their study revealed that urinary KIM-1 showed a progressive increase within 12 h following early acute renal ischemia. Other recent studies have shown that the detection of KIM-1 in kidney tissue and urine facilitates the early diagnosis of AKI and is a better indictor than Scr or serum BUN [12, 23, 24]. Our study also revealed that urinary KIM-1 levels were significantly higher in patients with severe burns than in the healthy control group and showed a significantly increasing trend with worsening burn severity. Urinary KIM-1 levels were also significantly higher in the moderate- and severe-burn-group patients with AKI than in those without AKI and were detected earlier than elevated Scr. Moreover, correlation analysis indicated that urinary KIM-1 levels were correlated with Scr, suggesting that urinary KIM-1 can be used as an early indicator of AKI.

IL-18 is a proinflammatory factor that promotes endogenous inflammatory processes. Patients with AKI as a complication of preeclampsia, contrast-induced nephropathy, and coronary angiography have significant increases in urinary IL-18 prior to the occurrence of increases in Scr and serum BUN [12, 13, 18]. In non-septic patients, urinary IL-18 was significantly elevated 2 days prior to the increase in Scr [12]. According to Risk, Injury, Failure, Loss of kidney function, and End-stage kidney disease (RIFLE) criteria, urinary IL-18 levels of AKI patients increased significantly 24–48 h prior to the occurrence of AKI, and urinary IL-18 levels were associated with the severity of AKI, thus suggesting that IL-18 is an important indicator for the prediction of AKI and subsequent mortality [25]. In fact, the sensitivity and specificity of urinary IL-18 levels for the diagnosis of AKI are greater than 90 % [6, 26]. In the present study, we also found that the urinary IL-18 levels of all three groups of burn patients were similar to those of the control group. However, urinary IL-18 levels were significantly higher in the AKI group than in non-AKI group, were detected earlier than increases in Scr, and were significantly correlated with Scr and serum BUN, thereby also suggesting that urinary IL-18 is a useful biomarker for predicting early AKI in burn-injury patients.

In the present study, ROC curve analysis of the ability of KIM-1 and IL-18 to predict AKI on admission demonstrated both KIM-1 and IL-18 to have high sensitivity and specificity, with AUCs of 0.846 and 0.819, respectively. However, the ability of the combination of urinary KIM-1 with IL-18 to predict AKI yielded an AUC of 0.904.

The results of our NRI and IDI analyses to estimate the predictive power of each biomarker also indicated that urinary KIM-1 combined with IL-18 may be a sensitive biomarker to predict early AKI.

Finally, we found urinary KIM-1 to have a significant positive correlation with the TBSA, TDBA, the presence of rhabdomyolysis, and APACHE II score, again suggesting that urinary KIM-1 may be associated with disease severity in burn patients, but this will require further investigation. Our results, which also suggest that urinary KIM-1 and IL-18 levels in the post-burn period may predict AKI, are consistent with recent reports demonstrating NGAL as an indicator of AKI in burn patients [10, 11].

There are some limitations to our study. We did not collect 6-, 12-, 24-, and 36-h urine and blood specimens in the post-burn period to observe the dynamic changes in these indicators; further research will be necessary to clarify these.

## Conclusions

Our results suggest that urinary KIM-1 and IL-18 may be used as early, sensitive indicators of AKI in patients with varying degrees of burns and may provide clinical clues that can be used for early prevention of AKI.

## Abbreviations

AKI: Acute kidney injury
Alb: Albumin
ALT: Alanine aminotransferase
APACHE II Score: Acute Physiology and Chronic Health Evaluation II Score
ARF: Acute renal failure
AUC: Area under the ROC curve
BUN: Blood urea nitrogen
CI: Confidence interval
CK: Creatine kinase
CRRT: Continuous renal replacement therapy
IDI: Integrated discrimination improvement
IL-18: Interleukin-18
KDIGO: Kidney Disease Improving Global Outcomes
KIM-1: Kidney injury molecule-1
NAG: N-acetyl-β-glucosidase
NGAL: Neutrophil gelatinase-associated lipocalin
NRI: Net reclassification improvement
ROC: Receiver operating characteristic
Scr: Serum creatinine
SE: Standard error
TDBA: Third-degree burn area
TBSA: Total burn surface area
